# Safety and immunogenicity of a SARS-CoV-2 mRNA vaccine (SYS6006) in minks, cats, blue foxes, and raccoon dogs

**DOI:** 10.3389/fcimb.2024.1468775

**Published:** 2024-09-19

**Authors:** Hong Huo, Jinming Wang, Chan Li, Shuang Xiao, Han Wang, Jinying Ge, Gongxun Zhong, Zhiyuan Wen, Chong Wang, Qiaoling Lang, Lili Chen, Zilong Wang, Jinliang Wang, Xijun Wang, Xijun He, Yuntao Guan, Lei Shuai, Zhigao Bu

**Affiliations:** ^1^ State Key Laboratory for Animal Disease Control and Prevention, Harbin Veterinary Research Institute, Chinese Academy of Agricultural Sciences, Harbin, China; ^2^ National High Containment Laboratory for Animal Diseases Control and Prevention, Harbin Veterinary Research Institute, Chinese Academy of Agricultural Sciences, Harbin, China; ^3^ CSPC Zhongqi Pharmaceutical Technology (Shijiazhuang) Co., Ltd., CSPC Pharmaceutical Group Co., Ltd., Shijiazhuang, Hebei, China; ^4^ Jiangsu Co-innovation Center for Prevention and Control of Important Animal Infectious Diseases and Zoonoses, Yangzhou University, Yangzhou, China

**Keywords:** SARS-CoV-2, mRNA vaccine, safety, neutralizing antibody, susceptible animals

## Abstract

Minks, cats, and some other species of carnivores are susceptible of the severe acute respiratory syndrome coronavirus 2 (SARS-CoV-2) and have a high risk of transmitting SARS-CoV-2 to humans. The development of animal vaccines can be an effective measure to protect animals against SARS-CoV-2 and reduce the potential risk of human infection. We previously developed a messenger ribonucleic acid (mRNA) vaccine SYS6006 that has been proven to be an efficient coronavirus disease 2019 (COVID-19) vaccine widely used in humans. Here, we further evaluated the safety and immunogenicity of SYS6006 as an animal COVID-19 vaccine candidate for SARS-CoV-2 susceptible animals or wild animals. SYS6006 was safe and immunogenic in mice and completely protected mice against mouse-adapted SARS-CoV-2 infection in the upper and lower respiratory tracts. SYS6006 was able to induce neutralizing antibodies against the SARS-CoV-2 wild-type, Delta, and Omicron BA.2 strain on day 7 after prime immunization, and two doses of immunization could enhance the neutralizing antibody responses and produce long-lasting potent antibodies for more than 8 months in minks and cats, blue foxes, and raccoon dogs, while all immunized animals had no abnormal clinical signs during immunization. These results provided here warrant further development of this safe and efficacious mRNA vaccine platform against animal COVID-19.

## Introduction

Severe acute respiratory syndrome coronavirus 2 (SARS-CoV-2) is a zoonotic pathogen, which caused the pandemic coronavirus disease 2019 (COVID-19) and more than 7 million human deaths worldwide (WHO, 2024). Vaccination against SARS-CoV-2 has been proven effective to contain the spread of the virus and reduce disease. More than 183 candidate vaccines against SARS-CoV-2 were tested in human clinical trials, and multiple vaccines have been approved for emergency application in efforts to control the spread of the virus, including inactivated vaccines, mRNA-, protein-, and DNA-, and viral-vector-based vaccines (He et al., 2023; WHO, 2023). However, SARS-CoV-2 continues to mutate and evolve into a lot of new variants of concern (VOCs) with increased breakthrough infections and are more infectious (Carabelli et al., 2023). Omicron and its sublineages have been shown to be susceptible to immune evasion and led to the weakened efficacy of approved vaccines (Cao et al., 2022; Zhou et al., 2023). Therefore, emerging VOCs have brought new challenges to the prevention of COVID-19, and updated vaccines are urgently needed to provide better protection against the Omicron variants or other highly mutated variants.

SARS-CoV-2 is believed to be animal-borne (Zhou et al., 2020). Although bats and pangolins were initially suspected to be reservoir and intermediate hosts of SARS-CoV-2, natural infection of farm, domestic, and wild animals by SARS-CoV-2 is indicative of its expanded host range, suggesting that many animal species could potentially play a role as reservoir or intermediate hosts for SARS-CoV-2 adaption to humans (Shi et al., 2020a; Islam et al., 2022; Nooruzzaman and Diel, 2023). Furthermore, accumulating evidence has emerged confirming that infected animals could transmit the virus to conspecifics or other species within their natural habitats, such as mink to mink, mink to cat, and among white-tailed deer, with transmission even to humans (Munnink et al., 2021; Sila et al., 2022; Caserta et al., 2023). Thus, protection of SARS-CoV-2-susceptible animals such as minks, cats, and other potentially susceptible animals is crucial to compensate for deficiencies of the animal epidemiological surveys and to prevent further zoonotic spillover.

mRNA vaccines are considered as a promising vaccine platform due to their safety, versatility, rapid development process, comparatively low production cost, and high efficacy (Pardi et al., 2018; Karam and Daoud, 2022). Results from clinical trials of the two mRNA vaccines, BNT162b2 and mRNA-1273, showed that their protective efficacy against SARS-CoV-2 infection were more than 90% after a two-dose immunization (Choi et al., 2021; Puranik et al., 2022). This vaccine platform has the immunological characteristics, combined with the defined composition and safety of inactivated or subunit vaccines. The SARS-CoV-2 mRNA vaccine, SYS6006, was developed by CSPC Zhongqi Pharmaceutical Technology Co., Ltd., (Shijiazhuang, Hebei, China) which combines the modified mRNA molecule of spike (S) protein of the SARS-CoV-2 wild-type (WT) strain with lipids to form lipid nanoparticles. SYS6006 showed humoral and cellular immune responses in non-human primates and had received an emergency use authorization (EUA) as a SARS-CoV-2 mRNA vaccine in China (Xu et al., 2023). Here, we evaluated the safety and immunogenicity of SYS6006 in two SARS-CoV-2-susceptible animals (minks and cats) and two SARS-CoV-2 potentially susceptible animals (blue foxes and raccoon dogs). The significant safety and immune responses of SYS6006 with a two-dose schedule immunization in SARS-CoV-2-susceptible and potentially susceptible animals support further clinical development of SYS6006 in animals.

## Materials and methods

### Cells and viruses

Vero E6 cells were grown in Dulbecco’s modified Eagle’s minimal essential medium (DMEM, Gibco, Waltham, MA, USA) supplemented with 10% fetal bovine serum (FBS, ExCell, Suzhou, Jiangshu, China). Mouse-adapted SARS-CoV-2/HRB26/human/2020/CHN (HRB26M, GISAID access no. EPI_ISL_459910) was generated by passaging the human patient isolate SARS-CoV-2/HRB26/human/2020/CHN (HRB26, GISAID access no. EPI_ISL_459909) in BALB/c mice and propagated in Vero E6 cells (Wang et al., 2020). A previously described VSVΔG-eGFP pseudovirus system was adapted and modified to construct VSVΔG*GFP/WTS, VSVΔG*GFP/δS and VSVΔG*GFP/BA2S (Wang et al., 2006). All viruses were stored at −70°C before use.

### SYS6006 production

The mRNA vaccine SYS6006 was produced from Zhongqi Pharmaceutical Technology Co., Ltd. of CSPC Pharmaceutical Group. The mRNA was synthesized from a linearized DNA template encoding modified S protein of the prototype SARS-CoV-2 strain and incorporated with a 5′ untranslated regions (UTR), a 3′ UTR and a poly A tail. The mRNA was then encapsulated to a lipid nanoparticle by a lipid mixture containing lipids, ethanol, ionizable lipids, DSPC, cholesterol, etc. through a micro-channel device. The formulations of lipid nanoparticle as the mRNA vaccine SYS6006 were tested for particle size, particle distribution, RNA concentration, encapsulation ratio, and mRNA sequence, and stored at −20°C before use.

### Mouse studies

Female BALB/c mice 6 weeks old (Vital River, Beijing, China) were randomly assigned to four groups (12/group). The four groups of mice were immunized intramuscularly (i.m.) at the gastrocnemius muscle with 5 μg, 10 μg, or 20 μg of SYS6006, or PBS (as the placebo group) in a 0.1-ml volume twice over 21-day intervals. The mice were observed daily for signs of disease and body weight changes for 21 days. Sera were collected for SARS-CoV-2 neutralizing antibody (NAb) titration at different time points after immunization. Six mice in each group were randomly selected to challenge intranasally (i.n.) with SARS-CoV-2 HRB26M (10^3.6^ PFU/0.05 ml/mouse) on day 14 post-boost immunization, and three mice of each group were sacrificed at 3 and 5 days post-challenge (dpc) for viral load quantification of turbinate and lung.

### Mink studies

Farmed minks from local farms 7–8 months old were confirmed to be serologically negative for SARS-CoV-2 before immunization and randomly assigned to two groups (12/group) by similar body weight and sex. The two groups of minks were immunized i.m. with 30 μg or 90 μg of SYS6006 in a 1-ml volume at the quadriceps femoris. At 3 weeks after the first dose, the minks were boosted with SYS6006 at the same dose and via the same route. The minks were observed daily for the mental state, dietary status, and signs of disease during the immunosurveillance period of up to 8 months. Serum samples were collected for SARS-CoV-2 NAb titration at different time points after immunization.

### Cat studies

Farmed minks from local farms 4–6 months old were confirmed to be serologically negative for SARS-CoV-2 before immunization and randomly assigned to two groups (12/group) by similar body weight and sex. The two groups of cats were immunized i.m. with 30 μg or 90 μg of SYS6006 and boosted at 3 weeks after the first dose using the same dose. The cats were observed daily for the mental state, dietary status, and signs of disease during the immunosurveillance period of up to 12 months. The cats were bled from the vein of the front leg at different time points after immunization for SARS-CoV-2 NAb titration.

### Fox and raccoon dog studies

Five 7–8-month-old farmed blue foxes and five 7–8-month-old farmed raccoon dogs from local farms were confirmed to be serologically negative for SARS-CoV-2 before immunization and immunized i.m. with 30 μg of SYS6006 twice over 21-day intervals. The foxes were observed daily for the mental state, dietary status, and signs of disease during the immunosurveillance period of up to 8 months. Serum samples were collected for SARS-CoV-2 NAb titration at different time points after immunization.

### Neutralization assays

Neutralizing antibody titers against SARS-CoV-2 WT, δ, or BA2 strain were determined using the pseudotyped recombinant vesicular stomatitis virus (VSV). The recombinant VSVs (VSVΔG*GFP/WTS, VSVΔG*GFP/δS, and VSVΔG*GFP/BA2S) can express GFP in cells, with an S protein of WT, δ, or BA2 strain into the envelope. Serum samples were serially diluted twofold in DMEM, starting at 1:8, with a volume of 0.05 ml. The diluted serum samples were mixed with 50 μl of DMEM containing 200 TCID_50_ of VSVΔG*GFP/WTS, VSVΔG*GFP/δS, and VSVΔG*GFP/BA2S and incubated at 37°C for 1 h. Subsequently, the virus–serum mixture was added to the Vero E6 cells in a 96-well plate. After 24 h of inoculation, GFP-expressing cells were counted under a fluorescence microscope, and the SARS-CoV-2 NAb titers were expressed as the reciprocal of the highest dilution of serum that showed at least a 50% reduction in the number of fluorescent cells compared to the cell control without serum sample.

### qPCR

The viral load was determined using quantitative real-time PCR (qPCR). Viral genomic RNA of SARS-CoV-2 was extracted by using the QIAamp vRNA Mini kit (Qiagen, Hilden, Germany). Reverse transcription was performed with the HiScript II Q RT SuperMix (Vazyme, Nanjing, China). qPCR was conducted to quantify the number of viral N gene RNA copies using the Applied Biosystems QuantStudio 5 Real-Time PCR System (Thermo, Waltham, MA, USA) with Premix Ex Taq (probe qPCR) (Takara, Dalian, China). The N gene-specific primers (forward, 5′- GGGGAACTTCTCCTGCTAGAAT-3′; reverse, 5′-CAGACATTTTGCTCTCAAGCTG-3′) and probe (5′-FAM-TTGCTGCTGCTTGACAGATT-TAMRA-3′) were utilized according to the information provided by the National Institute for Viral Disease Control and Prevention, China (http://nmdc.cn/nCoV). The amount of vRNA for the target SARS-CoV-2 N gene was normalized to a standard curve obtained by using a plasmid (pBluescriptIISK-N) containing the full-length cDNA of the SARS-CoV-2 N gene.

### Statistical analysis

The data were analyzed using GraphPad Prism software (GraphPad Software Inc., San Diego, CA). Statistical comparisons between the assay results were performed using a two-way ANOVA test and a two-tailed unpaired *t*-test. *p*-Values <0.05 were considered statistically significant.

### Ethics, facility, and biosafety statement

Animal experiments were approved by the Animal Ethics Committee of Harbin Veterinary Research Institute (HVRI) of the Chinese Academy of Agricultural Sciences (CAAS). The number of animals used in this study was determined by following the “minimum quantity principle” (Wang et al., 2020; Shuai et al., 2021). All experiments with infectious SARS-CoV-2 were performed within biosafety level 4 and animal biosafety level 4 facilities in the HVRI of the CAAS.

## Results

### SYS6006 meets the quality standards of the internal control of Zhongqi Pharmaceutical Technology Co., Ltd.

The candidate mRNA vaccine SYS6006 was produced by Zhongqi Pharmaceutical Technology Co., Ltd. as described before (Xu et al., 2023). All the key indicators of SYS6006 meet the quality standards through corresponding devices, with 96 nm of particle size, below 0.1 of particle distribution, 100 μm/ml of RNA concentration, 94% of encapsulation rate, and correct mRNA sequence, indicating that SYS6006 was well produced.

### SYS6006 is safe and induces strong humoral immune responses in mice

**Figure 1:**
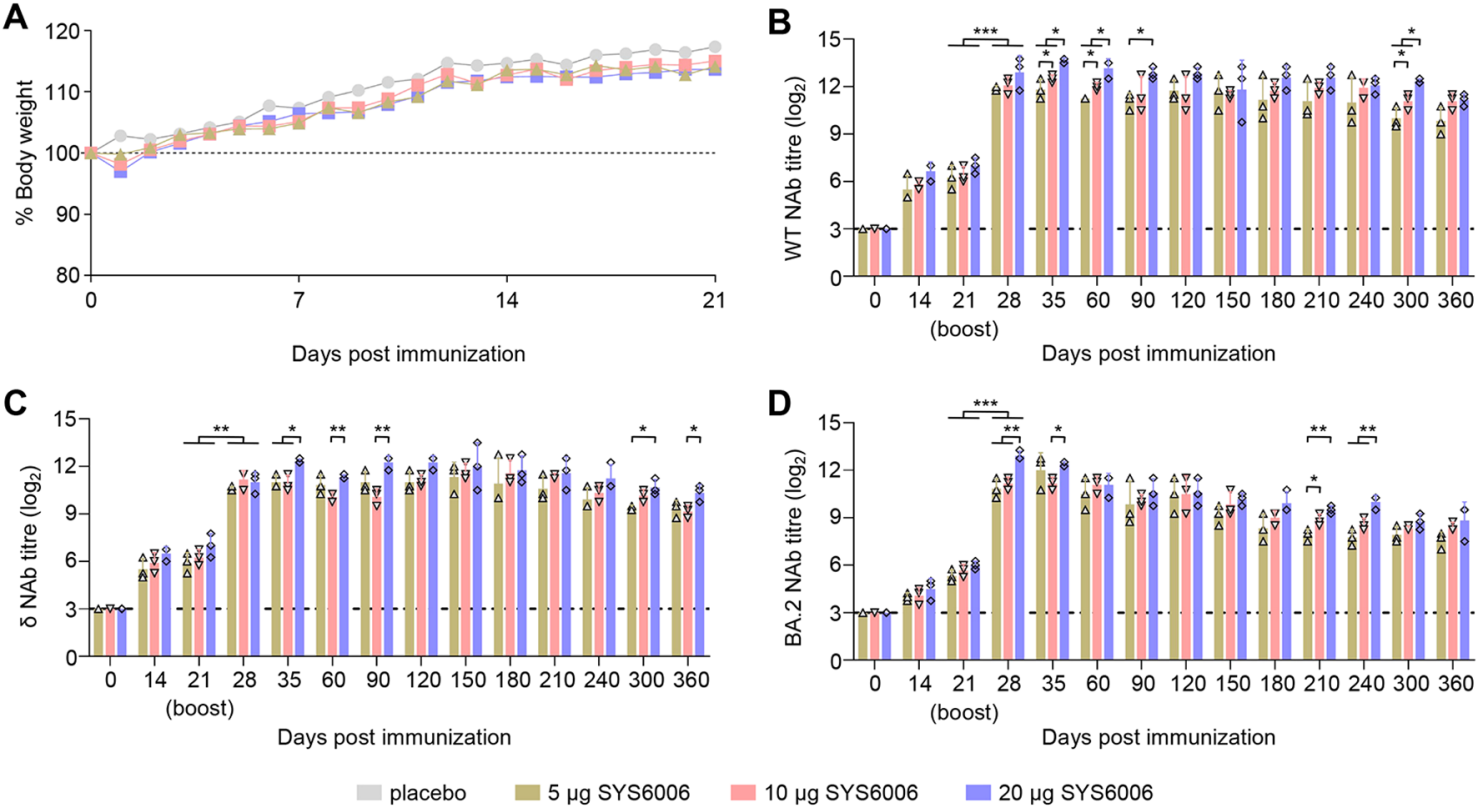
Immunization studies in mice. Mice were intramuscularly immunized with 5 μg, 10 μg, or 20 μg of SYS6006, or PBS (placebo) in a 0.1-ml volume twice over 21-day intervals. SARS-CoV-2 neutralizing antibody (NAb) were detected by a pseudotyped VSV-based neutralization assays. **(A)** Body weight changes of mice. **(B)** SARS-CoV-2 NAb against wild-type strain (WT) of immunized mice. **(C)** SARS-CoV-2 NAb against Delta strain (δ) of immunized mice. **(D)** SARS-CoV-2 NAb against Omicron BA.2 strain (BA.2) of immunized mice. The horizontal dashed lines indicate the lower limit of detection. Statistical significance was determined by using a two-way ANOVA test in weight changes, and a two-tailed unpaired *t*-test in NAb titers. *p < 0.05, **p < 0.01, ***p < 0.001.

The immunized mice were observed daily for signs of disease and body weight changes for 21 days. All immunized mice remained healthy and had no significant difference in the body weight changes between the various doses of SYS6006 and placebo during the observation period (**Figure 1A**). The SARS-CoV-2 NAb responses were evaluated in BALB/c mice at different time points after immunization. SYS6006 was able to stimulate the production of NAb against SARS-CoV-2 WT, δ, or BA2 strain at day 14 post-prime immunization. The NAb responses were boosted after the second dose, with the mean titer of 11.92–12.92 log2 against WT strain (**Figure 1B**), 10.58–11.17 log2 against δ variant (**Figure 1C**), and 10.83–12.92 log2 against BA.2 variant (**Figure 1D**) at day 28 post-prime immunization in 5 μg, 10 μg, or 20 μg immunized mice, and maintained at high levels sustained for the 360-day observation period. Compared to the 5- and 10-μg dosage, the 20-μg dosage induced higher NAb against the three strains. These results indicated that SYS6006 were safe and highly immunogenic in mice.

### SYS6006 protects mice from SARS-CoV-2 infection

**Figure 2:**
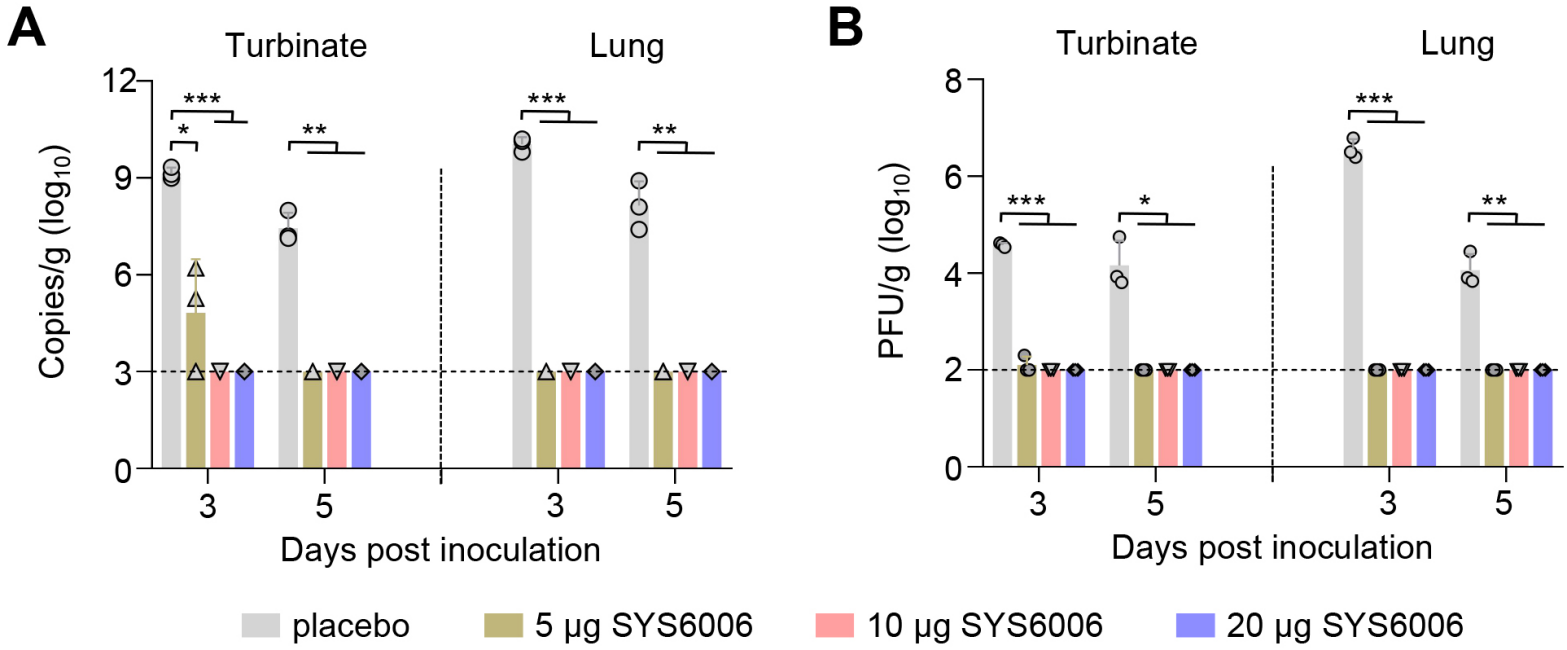
Protective efficacy of SYS6006 against SARS-CoV-2 infection in mice. The immunized mice were inoculated with SARS-CoV-2 HRB26M strain, and their turbinates and lungs were collected on day 3 and 5 post-inoculation. **(A)** viral RNA in turbinates and lungs of immunized mice. **(B)** Viral titers in turbinates and lungs of immunized mice. The horizontal dashed lines indicate the lower limit of detection. Statistical significance was determined by using a two-tailed unpaired *t*-test. *p < 0.05, **p < 0.01, ***p < 0.001.

After challenge, SARS-CoV-2 RNA was not detected in the turbinates and lungs of mice immunized with SYS6006 at doses of 10 μg and 20 μg at 3 (0/3) dpc and 5 (0/3) dpc. In 5 μg immunized mice, one mouse exhibited no detectable viral RNA in the turbinates on 3 dpc, while the other two had a significantly lower viral RNA copies in the turbinates compared to the placebo mice (**Figure 2A**). No infectious virus was detected in the turbinates and lungs of SYS6006-immunized mice on either 3 (0/3) dpc or 5 (0/3) dpc (**Figure 2B**). Viral RNA and infectious viruses were detected both in turbinates and lungs of all placebo mice on 3 (3/3) dpc and 5 (3/3) dpc, with a mean viral load of 9.15 lg copies/g in the turbinates and 10.05 lg copies/g in the lungs (**Figure 2A**), and a mean titer of 4.58 lg PFU/g in the turbinates and 6.57 lg PFU/g in the lungs (**Figure 2B**) on 3 dpc. These results indicated that SYS6006, administered with a two-dose schedule, provide complete protection against SARS-CoV-2 infection in both the upper respiratory tract and lungs of mice.

### SYS6006 is safe and induces SARS-CoV-2-specific humoral responses in minks

**Figure 3:**
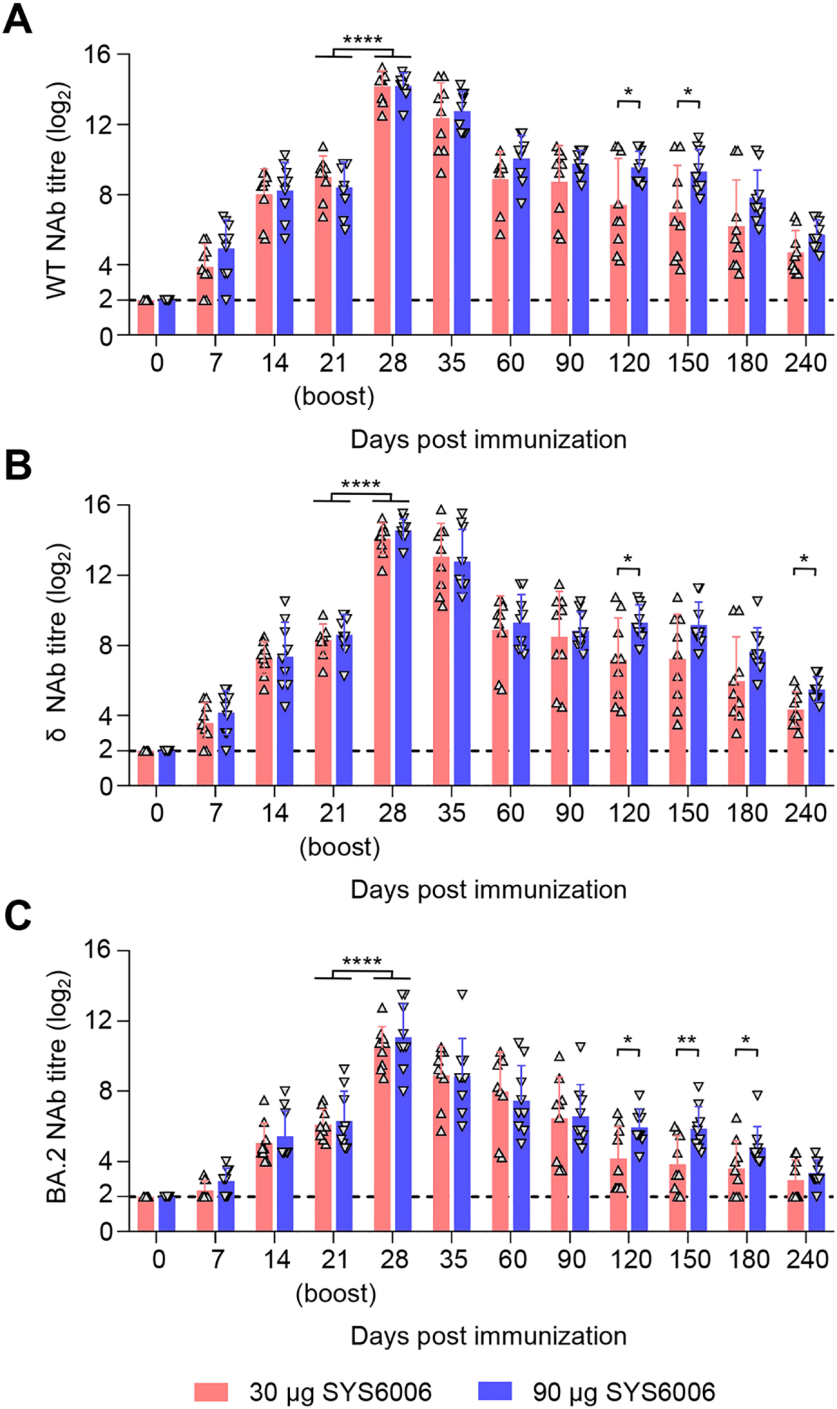
Immunogenicity studies in minks. Minks were intramuscularly immunized with 30 μg or 90 μg of SYS6006 twice over 21-day intervals in a 1-ml volume at the quadriceps femoris. SARS-CoV-2 NAb were detected. **(A)** SARS-CoV-2 NAb against wild-type strain (WT) of immunized minks. **(B)** SARS-CoV-2 NAb against Delta strain (δ) of immunized minks. **(C)** SARS-CoV-2 NAb against Omicron BA.2 strain (BA.2) of immunized minks. The horizontal dashed lines indicate the lower limit of detection. Statistical significance was determined by using a two-tailed unpaired *t*-test. *p < 0.05, **p < 0.01, ****p < 0.0001.

After a two-dose schedule immunization with the 30- and 90-μg dosage on 0–21 days, all minks showed no abnormal mental, diet, and other abnormal clinical conditions during the immunization observation period of up to 240 days. The immunization scheme with 30 μg of SYS6006 was able to induce the NAbs against SARS-CoV-2 WT, δ, and BA.2 strain at day 7 post-prime immunization, and the titers were significantly boosted after the second dose. The NAb titers against the WT (**Figure 3A**), δ (**Figure 3B**), and BA.2 (**Figure 3C**) strain of 30 μg dose-immunized minks significantly rose to peak at day 7 after boost immunization, with the mean titers of 14.16 log2, 14.08 log2, and 10.47 log2, and maintained at high levels sustained for the 240-day observation period. Compared to the 30-μg dosage, the 90-μg dosage induced higher NAb against the three strains during the 8-month observation period. SYS6006 appears to be safe and provide specific NAb against different SARS-CoV-2 variants in minks.

### SYS6006 is safe and induces SARS-CoV-2 specific humoral responses in cats

**Figure 4:**
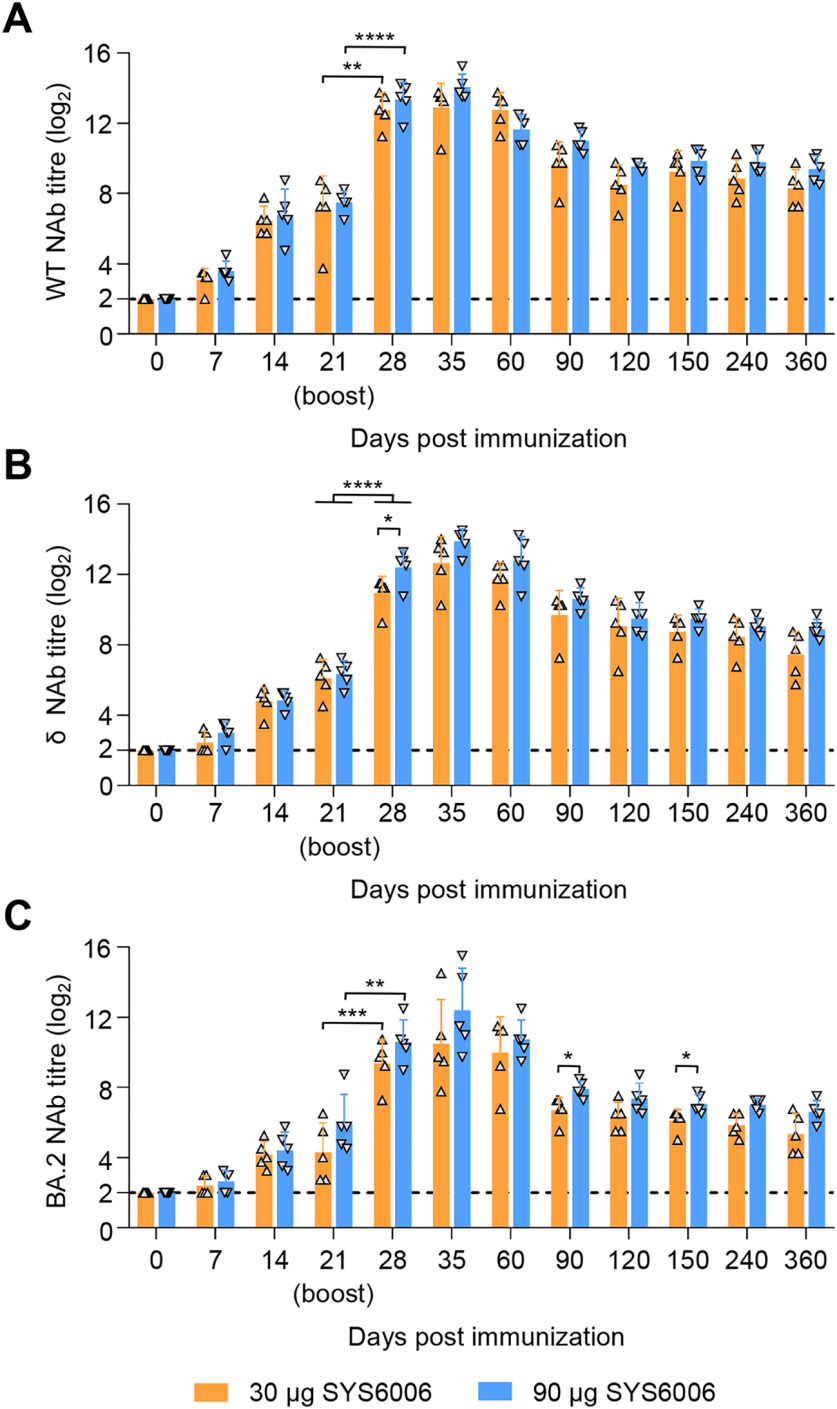
Immunogenicity studies in cats. Cats were intramuscularly immunized with 30 μg or 90 μg of SYS6006 twice over 21-day intervals in a 1-ml volume. SARS-CoV-2 NAb were detected. **(A)** SARS-CoV-2 NAb against wild-type strain (WT) of immunized cats. **(B)** SARS-CoV-2 NAb against Delta strain (δ) of immunized cats. **(C)** SARS-CoV-2 NAb against Omicron BA.2 strain (BA.2) of immunized cats. The horizontal dashed lines indicate the lower limit of detection. Statistical significance was determined by using a two-tailed unpaired *t*-test. *p < 0.05, **p < 0.01, ***p < 0.001, ****p < 0.0001.

After a two-dose schedule with the 30- and 90-μg dosage on 0–21 days, all cats showed no abnormal mental, diet, and other abnormal clinical conditions during the immunization observation period of up to 360 days. Similar to the immunogenic in minks, 30 μg or 90 μg of SYS6006 was able to induce specific NAb responses against the WT, δ, and BA.2 strain at day 7 post-prime immunization, and the titers were significantly rose to peak at day 14 after boost immunization. The mean NAb titers against the WT (**Figure 4A**), δ (**Figure 4B**), and BA.2 (**Figure 4C**) strain of 90 μg dose-immunized cats were 14.05 log2, 13.90 log2, and 12.40 log2, respectively, and higher than those of 30 μg dose-immunized cats with 12.9 log2, 12.65 log2, and 10.5 log2 at day 14 after boost immunization. The high level of SARS-CoV-2 NAb maintained for the 360-day observation period, with the mean titers against the three SARS-CoV-2 strains were 8.3 log2, 7.45 log2, and 5.35 log2 in 30 μg dose-immunized cats, and 9.4 log2, 8.9 log2, and 6.6 log2 in 90 μg doses-immunized cats, respectively (**Figures 4A–C**). SYS6006 appears to be safe and immunogenic in cats from a two-dose schedule.

### SYS6006 is safe and induces SARS-CoV-2-specific humoral responses in blue foxes

**Figure 5:**
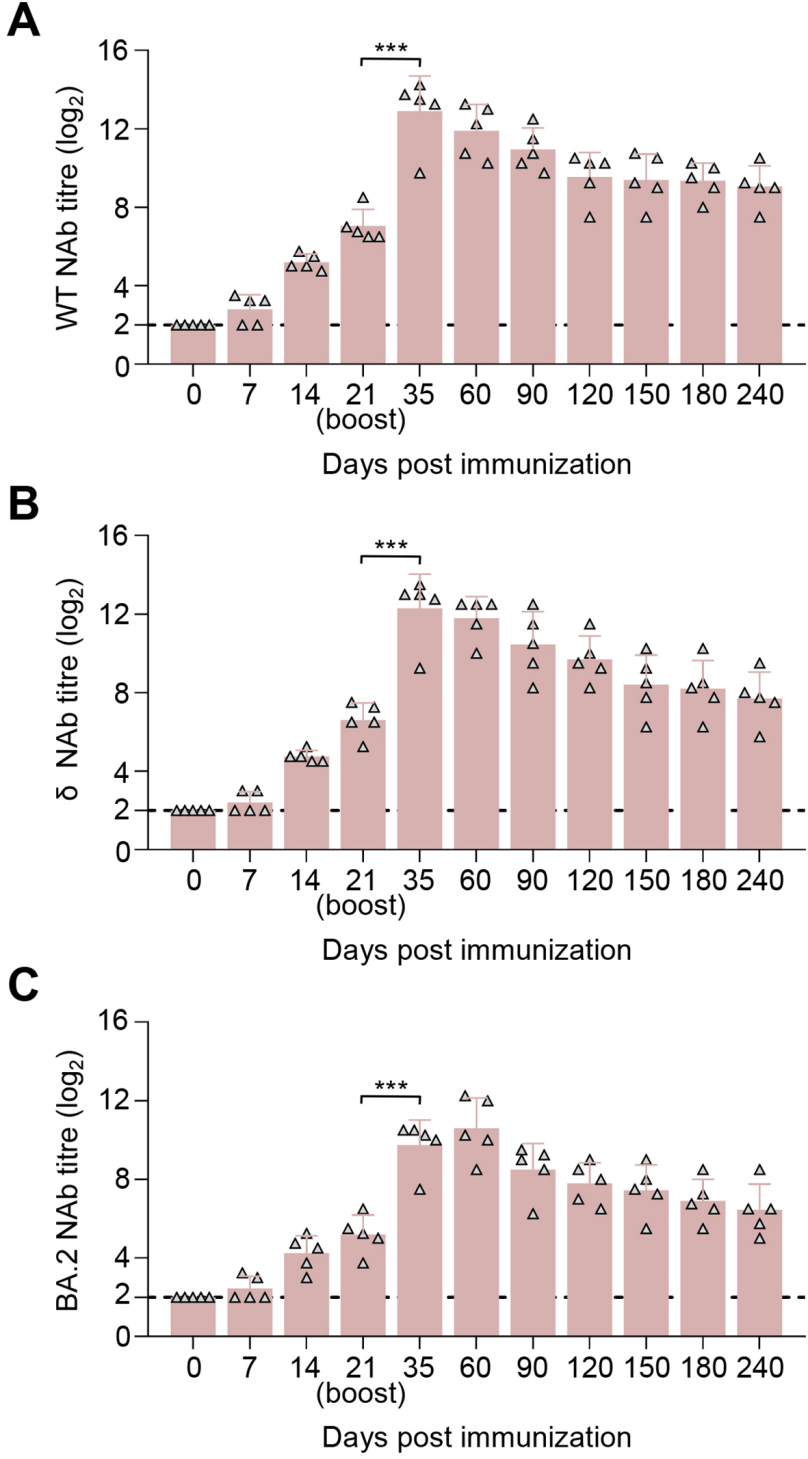
Immunogenicity studies in blue foxes. Five foxes were intramuscularly immunized with 30 μg of SYS6006 twice over 21-day intervals in a 1-ml volume. SARS-CoV-2 NAb were detected. **(A)** SARS-CoV-2 NAb against wild-type strain (WT) of immunized foxes. **(B)** SARS-CoV-2 NAb against Delta strain (δ) of immunized foxes. **(C)** SARS-CoV-2 NAb against Omicron BA.2 strain (BA.2) of immunized foxes. The horizontal dashed lines indicate the lower limit of detection. Statistical significance was determined by using a two-tailed unpaired *t*-test. ***p < 0.001.

The immunogenicity of SYS6006 was further investigated in foxes, which are potentially susceptible to SARS-CoV-2. After a two-dose schedule with the 30-μg dosage on 0–21 days, all foxes showed no abnormal mental, diet, and other abnormal clinical conditions during the immunization observation period of up to 240 days. SYS6006 of 30 μg was able to induce specific NAb responses against the WT (**Figure 5A**), δ (**Figure 5B**), and BA.2 (**Figure 5C**) strain at day 14 post-prime immunization. All five foxes exhibited high NAb responses at day 21 post-prime immunization, with the mean titers of 7.05 log2, 6.6 log2, and 5.2 log2 against the WT, δ, and BA.2 strain, respectively. The titers peaked at 12.9 log2, 12.3 log2, and 9.75 log2 at day 14 after boost immunization. During the 240-day observation period, the NAb responses against the three SARS-CoV-2 strains were detected and persisted, with the mean titers of 9.05 log2, 7.70 log2, and 6.45 log2 at day 240 after prime immunization (**Figures 5A–C**). These results indicated that SYS6006 was safe, highly immunogenic in foxes.

### SYS6006 is safe and induces SARS-CoV-2-specific humoral responses in racoon dogs

**Figure 6:**
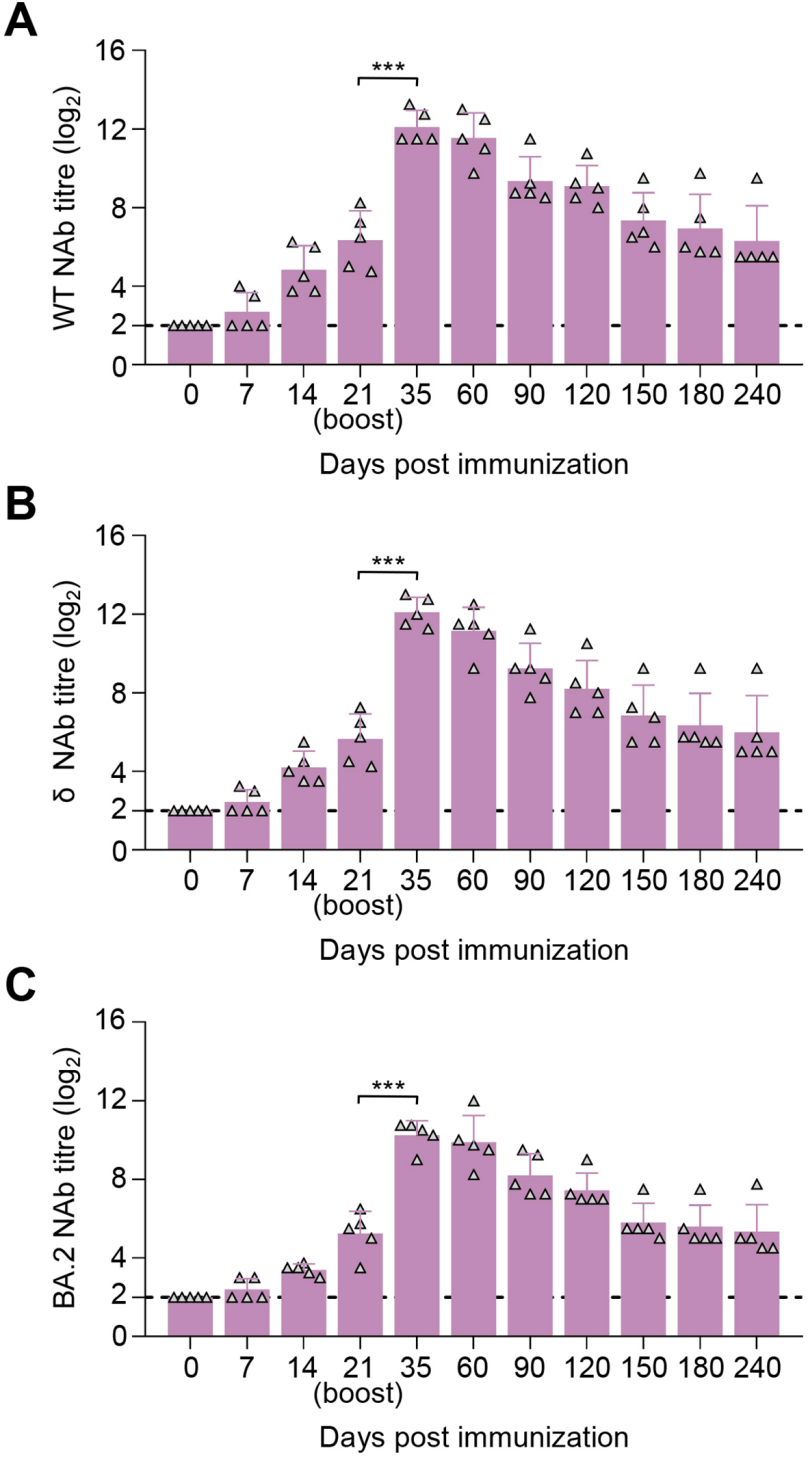
Immunogenicity studies in raccoon dogs. Five raccoon dogs were intramuscularly immunized with 30 μg of SYS6006 twice over 21-day intervals in a 1-ml volume. SARS-CoV-2 NAb were detected. **(A)** SARS-CoV-2 NAb against wild-type strain (WT) of immunized raccoon dogs. **(B)** SARS-CoV-2 NAb against Delta strain (δ) of immunized raccoon dogs. **(C)** SARS-CoV-2 NAb against Omicron BA.2 strain (BA.2) of immunized raccoon dogs. The horizontal dashed lines indicate the lower limit of detection. Statistical significance was determined by using a two-tailed unpaired *t*-test. ***p < 0.001.

Similar to the safety in foxes, all racoon dogs showed no abnormal mental, diet, and other abnormal clinical conditions during the 6-month immunization observation period after a two-dose schedule with the 30-μg dosage on 0–21 days. Racoon dogs also displayed immune responses against the WT (**Figure 6A**), δ (**Figure 6B**), and BA.2 (**Figure 6C**) strain, and all five racoon dogs exhibited high NAb responses at day 21 post-prime immunization, with the mean titers of 6.35 log2, 5.65 log2, and 5.25 log2 against the WT, δ, and BA.2 strain, respectively. The titers peaked at 12.1 log2, 12.1 log2, and 10.25 log2 at day 14 after boost immunization. During the 240-day observation period, the NAb responses against the three SARS-CoV-2 strains were detected and persisted, with the mean titers of 6.30 log2, 6.00 log2, and 5.35 log2 at day 240 after prime immunization (**Figures 6A–C**). These results indicated that SYS6006 were safe, highly immunogenic in racoon dogs.

## Discussion

Natural and experimental infections reveal the susceptibility of a variety of animal species to SARS-CoV-2 infection (Sailleau et al., 2020; Sit et al., 2020). Minks are highly susceptible to SARS-CoV-2, being classified as high-risk animals contracting the SARS-CoV-2 from infected humans and from other infected minks (Eckstrand et al., 2021; Koopmans, 2021; Lu et al., 2021). Minks may also be a reservoir for SARS-CoV-2, which could serve as a source for spillover into humans (Munnink et al., 2021). Companion animals, particularly cats, were reported to have relatively high outbreaks of SARS-CoV-2, which have increased the risk of SARS-CoV-2 widespread in humans (Zhang et al., 2020). COVID-19 vaccines for susceptible animals such as minks and cats can be an effective measure to protect them from SARS-CoV-2 infection and may in turn reduce the risk of human infection. Some veterinary COVID-19 vaccines, such as Carniva-Cov and Ancovax, have been developed in Russia and India for immunization in dogs, cats, minks, and foxes, respectively, and have produced relative antibody responses (Chavda et al., 2021; Choudhary et al., 2022). However, there is no approved animal COVID-19 vaccine in China. Therefore, it is necessary and urgent to develop animal COVID-19 vaccine, especially for susceptible animals such as minks and cats.

The application of COVID-19 vaccines has made great contributions to the control of COVID-19 infection. However, the virus continues to mutate, and the cross-protection effectiveness of existing vaccines is not high. Hence, the continuous updating of vaccines is needed. As SARS-CoV-2 continues to evolve with the emergence of new variants, the development of vaccine that provide broad-spectrum protection is critical to prevent mass infection caused by new variants. The previous study has shown that SYS6006 had demonstrated good safety, immunogenicity, and protective efficacy, and the bivalent vaccine SYS6006.32 (XBB.1.5/BQ.1) induced robust cross-neutralizing responses (Chen et al., 2023; Huang et al., 2024; Jin et al., 2024; Su et al., 2024). Both SYS6006 and SYS6006.32 have been urgently approved for human use and have proven to be safe and effective in humans. In this study, the immunization of SYS6006 completely blocked viral replication in the turbinates and lungs of mice, protecting mice from SARS-CoV-2 infection. As expected, SYS6006 also elicited highly long-term and cross-protective NAbs in minks and cats, which showed the potential for the development of animal COVID-19 vaccine. These findings were consistent with previous reports demonstrating that SYS6006 could induce broad protective antibody responses against SARS-CoV-2 in monkeys (Xu et al., 2023). In addition, minks and cats, immunized SYS6006 with two doses of high dosage (90 μg), did not show any adverse effects during the observation period of up to 8–12 months. These results provide strong support for the further evaluation of SYS6006 in clinical trials and suggest that it may be a promising candidate for broad protection against COVID-19 and its variants.

Many animals are susceptible to COVID-19, which involves 29 species of animals in Felidae, Canidae, and Mustelidae (Shi et al., 2020b; Nerpel et al., 2023). Due to the lack of surveillance data on SARS-CoV-2 infection in animals, there may be multiple potentially susceptible animals, and circulating COVID-19 strains may exist simultaneously in animal populations. Therefore, it is necessary to evaluate the efficacy of cross-protection to different strains of COVID-19 vaccine candidate in potentially susceptible wild animals or farmed animals. In this study, we chose foxes and raccoon dogs as SARS-CoV-2 potentially susceptible animals and expanded to evaluate the safety and efficacy of SYS6006 in the two species. As expected, SYS6006 was safe and induced robust SARS-CoV-2-specific cross-NAb maintaining at least for 8 months both in foxes and raccoon dogs after two dosages of immunization, suggesting that SYS6006 has the potential to be applied to potentially SARS-CoV-2 reservoirs.

To prevent and control the SARS-CoV-2 infection, multiple types of vaccines were developed by different technical pipelines, including inactivated vaccines, subunit vaccines, DNA vaccines, mRNA vaccines, and viral vectored vaccines. Compared with other types of vaccines, COVID-19 mRNA vaccines have the advantages of high safety and effectiveness, rapid immune response and long duration, response induced fast and long duration, no risk of nucleic acid integration, easy natural degradation *in vivo*, convenient update development, and stable large-scale production (Gregorio et al., 2024). Currently, several products have been authorized for emergency applications in humans, including Comirnaty^®^ (Pfizer), Spikevax (Modena), SYS6006 (CSPC), and SYS6006.32 (CSPC), which were recognized and accepted worldwide (Chalkias et al., 2022; Patel et al., 2022; Su et al., 2024). This study further proved that SYS6006 is equally safe and effective in minks, cats, foxes, raccoon dogs, and may be even in other potentially susceptible animals.

To date, there are limited results on the safety and stability of mRNA vaccines in animals, and more attention has been paid to the efficacy of the immune response associated with protection against SARS-CoV-2 infection (Feng et al., 2021; Gilbert et al., 2022). In addition, the long-term immune response to vaccines after immunization is of great importance and determines the clinical application strategy and protective efficacy (Slifka and Amanna, 2014; Lin et al., 2021). We showed that the antibody titers against different SARS-CoV-2 strains maintained high levels lasting over 12 months in mice and cats, over 8 months in minks, foxes, and raccoon dogs, indicating the persistence of humoral immune protection induced by two dosages of SYS6006.

## Conclusion

The evolution of SARS-CoV-2 is a challenge for vaccine-based strategies aimed at COVID-19 control. This report provides evidence that a two-dose immunization with an mRNA vaccine SYS6006 effectively protects against SARS-CoV-2 in multiple animal models. Based on the available data, the mRNA vaccine platform offers an option for both the development of vaccines and the preparedness for future pandemics.

## Data Availability

The datasets presented in this study can be found in online repositories. The names of the repository/repositories and accession number(s) can be found in the article/supplementary material.
